# Typhoid fever as a cause of opportunistic infection: case report

**DOI:** 10.1186/1471-2334-6-38

**Published:** 2006-02-27

**Authors:** Claudia Colomba, Laura Saporito, Laura Infurnari, Salvatore Tumminia, Lucina Titone

**Affiliations:** 1 Istituto di Patologia Infettiva e Virologia, Università di Palermo, Piazza Montalto 8, 90134 Palermo, Italy; 2 Unità Operativa di Malattie Infettive, Azienda Ospedaliera Universitaria Policlinico, Palermo, Italy

## Abstract

**Background:**

Typhoid fever is a systemic infection caused by the bacterium *Salmonella enterica *subspecies *enterica *serotype *typhi*, which is acquired by ingestion of contaminated food and water. Each year the disease affects at least 16 million persons world-wide, most of whom reside in the developing countries of Southeast Asia and Africa. In Italy the disease is uncommon with a greater number of cases in Southern regions than in Northern ones.

**Case presentation:**

We report on a 57-year-old Sri-Lankan male affected by typhoid fever, the onset of which was accompanied by oropharyngeal candidiasis. This clinical sign was due to a transient cell-mediated immunity depression (CD4+ cell count was 130 cells/mm^3^) probably caused by *Salmonella typhi *infection. Human immunodeficiency virus infection was ruled out. Diagnosis of typhoid fever was made by the isolation of *Salmonella typhi *from two consecutive blood cultures. The patient recovered after a ten days therapy with ciprofloxacin and his CD4+ cell count improved gradually until normalization within 3 weeks.

**Conclusion:**

Our patient is the first reported case of typhoid fever associated with oropharyngeal candidiasis. This finding suggests a close correlation between *Salmonella typhi *infection and transitory immunodepression.

## Background

Typhoid fever is a systemic infection caused by the bacterium *Salmonella enterica *subspecies *enterica *serotype *Typhi*, which is acquired by ingestion of contaminated food and water. Each year the disease affects at least 16 million persons world-wide, most of whom reside in the developing countries of Southeast Asia and Africa [1]. Typhoid fever is uncommon in industrialised regions such as the USA, Canada, Europe, Australia and Japan and new cases of the disease in these countries are related to travel to developing countries [2,3]. Based on such data, the public health authorities in most industrialised countries recommend vaccination against typhoid fever for travellers to the developing world, where sanitary conditions are poor [4]. Italy is a low endemicity country for typhoid fever and a greater number of cases occur in Southern regions than in Northern ones. Sicily is the second region of our country for incidence, with a mean of 100 annual cases in the last five years.

Although typhoid fever is classically described as an acute illness with fever and abdominal tenderness, the symptoms are non specific and may be insidious in onset [5]. The diagnosis of enteric fever should be seriously considered in the evaluation of travellers who return from tropical and subtropical areas with fever. Mortality rates associated with typhoid fever vary from region to region, with the highest reported from Indonesia, Nigeria, and India [6].

We report on an imported case of typhoid fever, the onset of which was accompanied by oropharyngeal candidiasis.

## Case presentation

In November 2004, a 57-year-old Sri-Lankan male turned up at the emergency department of our hospital. The patient had been living in Italy for 14 years, when he went back home to Sri-Lanka. He was there for 2 months, before returning to Italy where, few days later, he began suffering from fever, malaise, headache and non productive cough. He turned up at the emergency department of our hospital after 10 days from the beginning of symptoms.

On the basis of anamnestic data no history of drinking, smoking and illicit drug abuse was reported. Moreover, in his medical history there was no evidence of serious illness, such as diabetes mellitus or human immunodeficiency virus (HIV) infection. The patient had not been using proton pump inhibitors of stomach acid tablets, neither oral glycocorticosteroids or inhalation corticosteroids. Moreover, he did not need to take any antibiotics during his stay in Sri-Lanka.

The patient had a blood pressure of 180/100 mm Hg, a heart rate of 120 beats/min, a respiratory rate of 16 breaths/min, and a temperature of 38.9°C. On chest examination bilateral scattered rhonchi and mild rales were audible at the base of both the man's lungs. A chest radiograph revealed accentuation of the pulmonary reticulum, more marked on the right, but no clear signs of consolidation. The rest of the examination was notable for a minimally distended abdomen which was diffusely tender. Laboratory examination revealed a white blood cells (WBC) count of 6,400 cells/mm^3 ^(normal range 4,000–10,000 cells/ mm^3^), a haemoglobin level of 15 g/dl (12–17 g/dl), a platelet count of 189,000 cells/mm^3 ^(150,000 – 450,000 cells/mm^3^), an aspartate amino-transferase level of 71 U/L, an alanine aminotransferase level of 98 U/L; blood glucose level, serum electrolyte concentrations and renal function tests were within normal limits. A presumptive diagnosis of bronchitis was made, and the patient was transferred to our infectious disease department.

At the time of admission, the patient was persistently febrile (39°C). On physical examination we noted dry skin and oral candidiasis characterized by creamy white, curdlike patches on the tongue; this clinical picture was so typical that it did not need a culture to confirm the diagnosis of oral candidiasis. His abdomen was diffusely tender to palpation and without recognizable hepatosplenomegaly. Cultures of blood and respiratory specimens were performed. Thick and thin blood smears for malaria were obtained.

Treatment with topical nystatin and empirical therapy with ciprofloxacin (500 mg iv every 12 hours) were started. Because of the oro pharyngeal candidiasis, lymphocyte count was taken, the absolute number of lymphocytes was 562 cells/ mm^3^. The CD4+ cell count was 130 cells/mm^3^while the CD8+ was 240; as a second step an HIV test (ELISA) was performed and was negative. To rule out a very recent infection a qualitative HIV PCR was performed and HIV RNA was undetectable. On the 3rd day of hospitalisation, a *Salmonella *species identified as *S. typhi *was isolated from the two consecutive blood cultures obtained on the day of admission. Isolates were susceptible to ampicillin, cotrimoxazole, chloramphenicol, cefotaxime, ceftriaxone, and ciprofloxacin with the standard disk diffusion susceptibility testing method. The patient continued ciprofloxacin for a total of 10 days; he became afebrile after 3 days of therapy and his oral candidiasis improved slowly with nistatin. The patient recovered and was discharged from the hospital on the 10th day. At that time, his CD4+ cell count was 622 cells/mm^3^.

On the 20th day after discharge and in the 3rd month of follow-up, the patient was symptom free and his CD4+ cell count was raised to 1,080 and 1,200 cells/mm^3 ^respectively.

## Conclusion

In Italy, typhoid fever has become an uncommon disease occurring mainly as a sporadic disease in patients of any age and gender, and at any time of the year. The prevalence rate of typhoid fever has markedly decreased, from nearly 10,000 documented cases annually in the 1970s to a few hundred cases annually in the 2000s [7,8]. This data suggest that Italy is still a low endemicity country for typhoid fever, because of the presence of a moderate number of autochthonous cases mainly reported in the Southern regions of our country [9]. Nevertheless, *S. typhi *infection is one of the major causes of fever in patients admitted in hospital after returning from the tropics. Together with hepatitis A it represents the cause of a significant number of hospitalizations that pretravel vaccination strategies could have prevented [10].

Recently many strains of multidrug resistant (MDR) *S. typhi *have been identified worldwide [11-14]; in Italy an epidemiologic study conducted between 1980 and 1996 showed that their spread was until now quite low [15]. However an accurate and continuous surveillance is necessary in order to quickly identify MDR *S. typhi *strains and prevent their spread.

We report on an imported case of typhoid fever as previously described in other developed countries [16].

Patients with typhoid fever usually have a history of prolonged fever, headache and abdominal discomfort. There are no distinctive clinical features, and definitive diagnosis requires isolation of *S*. *typhi *from blood, bone marrow, stool, or urine cultures [5]. Our patient had fever with upper airway symptoms and showed typical lesions observed in oral candidiasis.

To date, oropharyngeal candidiasis is an opportunistic infection that generally develops in patients who have been using inhaled steroids over a long period of time, and those with cancer and AIDS [17,18]. Because our patient did not belong to any of the three categories of patients reported above, we performed an HIV test to rule out any recent HIV infection; the result was negative [19]. We noticed that the CD4+ cell count was low in the acute phase of disease and improved gradually until normalization within 3 weeks. These findings support the hypothesis that *S. typhi *could lead to a progressive reduction of CD4+ T helper cell population as already demonstrated by a previous study performed on mice infected with *S. typhimurium *[20].

In conclusion, to our knowledge our patient is the first reported case of typhoid fever the onset of which was accompanied by oropharyngeal candidiasis, suggesting a close correlation between *S. typhi *infection and transitory immunodepression.

## Competing interests

The author(s) declare that they have no competing interests.

## Authors' contributions

All authors contributed equally to this work

## Pre-publication history

The pre-publication history for this paper can be accessed here:


